# Psychological Factors Influencing Pain Perception and Experience in Women Undergoing Mammography: Protocol for a Systematic Review

**DOI:** 10.2196/77118

**Published:** 2025-11-12

**Authors:** Irene Neophytou, George Charalambous, Eleni Jelastopulu

**Affiliations:** 1 Department of Health Sciences, Frederick University, Gianni Freiderikou 7, Nicosia, 1036, Cyprus, 357 97442553; 2 University of Patras, Patras, Greece

**Keywords:** screening mammography, breast cancer screening, psychological factor, anxiety, pain catastrophizing, pain

## Abstract

**Background:**

Breast cancer is the most frequently diagnosed cancer among women worldwide and a leading cause of cancer-related mortality. Mammographic screening significantly improves the early detection and survival rates. However, the pain and discomfort experienced during mammography, primarily due to breast compression, can serve as major deterrents to participation in routine screening programs. Psychological factors such as anxiety, fear, and pain catastrophizing have been shown to influence pain perception and experience during mammography. These factors may affect women’s decisions to participate in or avoid screening, undermining public health efforts for early detection.

**Objective:**

This study aims to synthesize the scientific literature on the psychological factors influencing pain perception and experience in women undergoing mammography.

**Methods:**

This systematic review protocol is in accordance with the 2015 PRISMA-P (Preferred Reporting Items for Systematic Reviews and Meta-Analysis Protocols) guidelines. Eligible studies will include randomized controlled trials and observational designs that examine psychological factors—such as anxiety, catastrophizing, and related constructs—in relation to pain perception and experience among women undergoing screening or diagnostic mammography. The primary outcome is women’s perception and experience of pain during mammography, and the role of psychological factors may influence it, while secondary outcomes include pain intensity and pain-related distress, measured with validated pain scales or self-reported questionnaires. There will be no restriction on publication year, but only peer-reviewed, full-text articles in English will be included. Gray literature will be excluded. A systematic search will be conducted in PubMed, Scopus, and PsycINFO using database-specific strategies with keywords and Boolean operators; reference lists of included studies will also be screened. Study selection and data extraction will be performed independently by two reviewers. Risk of bias will be assessed using the Joanna Briggs Institute critical appraisal tools. Data will be synthesized narratively, with thematic grouping of psychological factors and tabulation of study characteristics. Due to anticipated heterogeneity across populations, study designs, and outcome measures, a meta-analysis will not be feasible; instead, greater interpretive weight will be given to findings from studies judged to have a lower risk of bias.

**Results:**

The database search has been completed in September 2025. Data extraction and organization into summary tables are scheduled to be finished by December 2025, followed by a narrative synthesis of findings. The systematic review manuscript is planned for submission to a peer-reviewed journal in January 2026.

**Conclusions:**

This protocol outlines the first systematic review to comprehensively investigate women’s perception and experience of pain during mammography and the psychological factors, such as anxiety, depression, fear, and coping strategies, that may influence it. The review aims to generate evidence-based insights that will inform clinical practice and guide the development of targeted interventions designed to reduce discomfort, improve screening experiences, and increase participation in breast cancer screening programs. The authors also plan to disseminate the findings through publication in a peer-reviewed journal.

## Introduction

### Background

Breast cancer remains a significant global public health issue and is the most common malignancy among women. It ranks among the top three most prevalent cancers worldwide, alongside lung and colorectal cancer [1,2]. In 2022, approximately 2.3 million women were diagnosed with breast cancer, resulting in 670,000 deaths globally [3]. The annual incidence continues to rise by 0.5%, and projections suggest that cases will surpass 3 million by 2040. While mortality has significantly declined since the 1980s due to advances in early detection and treatment, this decline has slowed in recent years. Breast cancer remains the leading cause of cancer-related death in women. African American women show disproportionately high mortality rates, and countries such as China are witnessing a sharp increase in incidence [4].

Screening programs are designed to detect the disease at an early stage and ultimately reduce breast cancer-related morbidity and mortality [5-7]. Population-based screening has substantially decreased breast cancer mortality rates in high-income countries. However, such programs require robust health system infrastructure, significant resource investment, and equitable service delivery. These prerequisites often present barriers in low- and middle-income countries, where healthcare systems may be fragmented, under-resourced, and understaffed, making quality screening and follow-up services difficult. Consequently, many low- and middle-income countries lack effective population-based breast cancer screening programs or fail to achieve the desired public health impact [8]. In countries such as India and those across Sub-Saharan Africa, implementation is often hindered by limited financial and logistical resources [9,10].

Mammographic screening is widely available and is regarded as one of the most effective methods for the early detection and prevention of breast cancer before the onset of clinical symptoms [6,11]. This has led many European countries, including the United Kingdom, Spain, and Germany, to establish population-based mammography screening programs [12]. For women at average risk, most guidelines recommend screening between the ages of 40 and 74 years, with the 50‐69 age group typically considered the optimal target for preventive screening [13].

Mammography utilizes X-rays to detect early signs of breast cancer. The standard protocol includes two views: craniocaudal and mediolateral oblique, which involve pulling and separating the breast tissue from the chest wall. During the procedure, the breast is compressed between two plates to immobilize the tissue and reduce its thickness, thereby minimizing radiation exposure and improving image quality [14,15].

Pain during medical procedures is widely recognized as a complex experience influenced not only by physiological factors but also by psychological, emotional, and cognitive elements [16]. Research across various clinical settings, such as gynecological examinations, colonoscopies, and dental work, has shown that psychological factors—particularly anxiety, fear, past experiences/expectations, and maladaptive coping strategies—can heighten pain perception and reduce adherence to preventive screenings [17-19]. In breast imaging, these psychological aspects are essential, as mammography requires repeated participation to meet public health goals.

While some women experience only mild discomfort during mammography, others describe the procedure as painful or distressing. Compression of the breast can cause pain during and after the examination, which has been shown to deter women from attending subsequent screening appointments, despite the known benefits of early detection [15,20]. A systematic review reported that between 25% and 46% of women cited pain during compression as the primary reason for avoiding future mammography [21].

Psychological factors such as situational anxiety and fear of pain play a significant role in pain perception during mammography [22,23]. Anxiety is the most reported emotional reaction to mammography, and fear may act as a barrier to participation [24]. General cancer-related anxiety may facilitate screening adherence, particularly when women believe in the effectiveness of mammography and have the necessary resources to access it [25]. However, anticipatory anxiety specifically related to the procedure itself may reduce compliance, as women seek to avoid the examination to alleviate their distress [26-28].

Numerous studies have demonstrated that pain catastrophizing—exaggerated negative thoughts and feelings about pain—contributes to higher pain intensity, emotional distress, and avoidance behavior [29,30]. Catastrophic thoughts regarding mammographic pain (eg, “the pain will be overwhelming”) can significantly affect screening adherence, particularly among breast cancer survivors. The cognitive-behavioral model of anxiety highlights how such thoughts can fuel avoidance of anxiety-provoking situations [31,32].

In the context of breast cancer, catastrophizing has been studied regarding screening adherence [33], persistent breast pain and mammography-related pain among survivors [34,35], and cancer worry among first-degree relatives of survivors [36]. Although limited, evidence from studies [37] suggests that healthy women with higher levels of catastrophizing and self-reassurance tendencies report greater pain during mammography.

However, the available findings are not entirely consistent. While several studies report significant associations between psychological factors—such as anxiety, catastrophizing, or depression—and mammography-related pain, others describe weaker or non-significant relationships [37-40]. This inconsistency may be due to differences in study design, the populations studied, and the variables evaluated.

Despite these insights, the available evidence remains fragmented and partly contradictory, as most studies have focused on individual psychological factors or specific subgroups. To date, no systematic review has synthesized the literature on psychological determinants of pain perception during mammography. This represents a significant knowledge gap, as a comprehensive synthesis could clarify the extent to which psychological factors contribute to mammography-related pain and influence women’s adherence to screening. Addressing this gap is essential for designing targeted interventions aimed at reducing discomfort, improving women’s experiences, and enhancing participation in breast cancer screening programs.

### Objective

This study aims to synthesize the scientific literature on the psychological factors influencing pain perception and experience in women undergoing mammography.

### Review Questions

The primary question was, “Is there an association between underlying psychological factors in women undergoing mammography and the experience of pain?” The secondary question was, “What is the intensity of pain experienced by women during mammography, as assessed through validated questionnaires?”

## Methods

### Overview

This protocol follows the 2015 PRISMA-P (Preferred Reporting Items for Systematic Review and Meta-Analysis for Protocols) checklist, and it is intended that this systematic review will contain the items of the PRISMA (Preferred Reporting Items for Systematic Reviews and Meta-Analyses) guidelines, 2020 version. This systematic review is registered with PROSPERO (ID: CRD420251117801). This protocol does not amend a previously completed or published protocol. Any necessary amendments made during the review process will be documented in the final report and transparently reported in the published manuscript.

### Eligibility Criteria

The selection of studies is based on the eligibility criteria given below.

#### Population

We will include studies involving women undergoing screening or diagnostic mammography. Studies that include patients with breast augmentation or breast reconstruction following mastectomy will also be eligible. All races and ethnicities of women reported in the included studies will be considered for inclusion. No exclusion criteria will be applied based on prior cancer history or comorbidities.

#### Exposure of Interest

Studies will be included if they examine psychological factors such as anxiety, fear of pain, and pain catastrophizing, or any combination of these exposures. Any additional or emerging psychological constructs related to pain perception during mammography identified in the literature will also be considered eligible for inclusion.

#### Outcomes of Interest

The primary outcome of interest is the perception and experience of pain among women undergoing mammography. Specifically, the review will assess whether psychological factors influence how pain is perceived and experienced during the procedure.

Secondary outcomes will include the level of pain experienced during or after mammography, assessed through validated pain scales or self-reported questionnaires, and whether this pain level is associated with specific psychological factors.

#### Type of Studies

All randomized control trials and comparative observational studies, including prospective and retrospective cohort studies, case-control studies, and cross-sectional studies, will be eligible. Reviews, including narrative and systematic reviews, conference abstracts, case series, case reports, editorials, and commentaries, will be excluded.

#### Language and Publication Status

We will include publications in the English language, and we will consist of studies reported as full text.

#### Years Considered

There will be no publication date restrictions.

#### Information Sources

A systematic literature search will be performed in the PubMed, Scopus, and PsycINFO databases using text words related to psychological factors and perception of pain undergoing mammography. Based on the references of the included studies, we will identify further relevant studies. Gray literature (eg, dissertations, conference abstracts, unpublished reports) will be excluded to ensure that only peer-reviewed studies of established methodological quality, with sufficient methodological and statistical details, are included in the synthesis.

#### Search Strategy

The project team will develop the search strategy. We will perform a preliminary search in the PubMed database to identify the first set of publications suitable for inclusion. We will identify additional keywords to build a final search strategy. This final search strategy will be reused for the PubMed database and adapted for the abovementioned further databases. The database searches will use keywords and subject terms connected by Boolean operators (OR and AND) to create specific search strategies for each database. In Textbox 1, the complete search strategy for the PubMed (MEDLINE) database is presented. From their inception, we will search all databases, restricting our search to English-language publications or publication status. We will check the reference lists of all included studies and any relevant systematic reviews identified for additional references to trials.

Textbox 1. **Search strategy for PubMed.**

((mammograph* OR "screening mammograph*" OR mammogram* OR "breast cancer screening" OR "mammographic screening")) AND (("Psychological factor*" OR anxiety OR stress OR fear OR "fear of pain" OR pain OR catastrophizing OR "pain catastrophization" OR depression OR "coping strateg*" OR neuroticism OR extraversion))) AND ((pain OR soreness OR tenderness).)

#### Data Management

Literature search results will be stored in Mendeley Reference Manager for duplicate removal and reference organization.

#### Selection Process

After removing duplicates, two independent reviewers will independently assess titles and abstracts of all publications from the databases. Studies that do not meet the inclusion criteria are excluded from further consideration. Any inconsistency between the researchers will be solved by discussion, and the reasons for each excluded study will be recorded. A third reviewer will be consulted to make the final decision if consensus cannot be reached. Full texts of the remaining studies will be treated in the same way. The selection procedure will be documented in a PRISMA flow diagram.

#### Data Collection Process

The review team will use a standardized data charting form to extract the data from the included studies. The template will be piloted on a subsample of three studies by two reviewers before use to ensure consistency, where any adaptations or refinements made will be reported. All extracted data will be verified for accuracy by a second reviewer. Any disagreements will be resolved by consensus or, where necessary, a third reviewer.

#### Data Items

From each included study, the following data will be extracted in a table: study details (eg, authors, year of publication, country of study, study design, and sample size), participants characteristics (eg, age, menopausal status, clinical characteristics, history of mammography, other relevant demographics), psychological factors and assessment tools (eg, anxiety, depression such as State-Trait Anxiety Inventory, Hospital Anxiety and Depression Scale, or other scales), pain outcome measures (eg, visual analog scale, or other validated tools), and main outcomes (eg, associations between psychological factors and pain during mammography, proportion of women reporting pain). This list is not exhaustive and may be refined further during the review process. Any discrepancies during data extraction will be resolved by discussion between the reviewers.

#### Risk of Bias in Individual Studies

The quality assessment of the included studies will be conducted using the Joanna Briggs Institute (JBI) checklist [41]. These tools offer checklists for various study designs. The assessment will address potential sources of bias in each study, such as participant selection, reliability of outcome measurements, appropriateness of the statistical analysis, and potential selective reporting of results. Responses to the questions are categorized as “yes,” “no,” “unclear,” and “not applicable.” A study is considered “unclear” if it does not provide explicit information for a specific question. If a question is irrelevant to the study’s context, it is marked as “not applicable.” Two reviewers will perform this process independently. Subsequently, the agreement between reviewers will be evaluated, and in cases of disagreement, consensus will be reached. No studies will be excluded based on quality; however, the appraisal results will be reported to allow readers to assess the credibility of the findings. Instead, the findings will inform the synthesis, with results from studies at higher risk of bias receiving less interpretative weight in the narrative synthesis. We will present stratified summaries of evidence according to risk of bias level (low, moderate, and high). The results of this quality assessment will be displayed in tabular form in the systematic review.

### Data Synthesis

In this review, the search is expected to reveal heterogeneous studies in the population, outcome measures, psychological constructs, and study designs. This diversity prevents meaningful pooling or meta-analysis of results, as combining heterogeneous effect estimates would obscure rather than clarify relationships. Therefore, the meta-analysis of study findings is not an objective of this review. Nevertheless, whenever possible, descriptive quantitative data (such as mean pain scores, prevalence of moderate-to-severe pain, or correlation coefficients) will be tabulated to provide a quantitative overview; however, the interpretation will mainly remain narrative. Data synthesis will take the form of a structured narrative synthesis of the included studies. The synthesis will be conducted in line with the framework proposed by Popay et al [42], which provides a systematic, transparent, and reproducible approach for narrative synthesis in systematic reviews. The defining characteristic of this approach is that it adopts a textual and thematic method to summarize and interpret findings in a structured manner. The synthesis will be organized around two main themes: (1) findings on the association between psychological factors and the experience of pain during mammography will be synthesized; (2) findings on the intensity of pain experienced by women during mammography will be collated from all studies. After findings on the association between psychological factors and the experience of pain during mammography will be synthesized, the extracted quantitative results (eg, correlation coefficients, *P* values, regression outcomes, or categorical findings such as “significant association” or “no association”) will first be categorized by the direction and strength of the association (positive, negative, or non-significant). These quantitative findings will then be qualitatively coded into broader thematic statements (eg, “anxiety amplifies pain perception,” “adaptive coping mitigates pain,” or “previous painful experience predicts greater pain”). This process will enable the integration of diverse statistical results into clear thematic patterns across studies. (2) Findings on the intensity of pain experienced by women during mammography will be collated from all studies. Reported pain outcomes (eg, visual analog scale scores, categorical pain levels, or prevalence rates) will be grouped according to each study’s measurement scale and reporting format. Descriptive summaries will be presented where available, and narrative patterns will be identified (eg, “higher intensity of pain among women, greater anxiety’’). The synthesis will highlight similarities and differences in reported pain intensity across studies. The results of the JBI quality appraisal will be integrated into the synthesis to aid interpretation, with greater weight given to findings from studies assessed as higher quality. Any discrepancies or variations across studies will be explicitly reported, and areas of consistency will be highlighted to provide a comprehensive overview of the evidence.

### Ethical Considerations

Ethical clearance is not applicable as the present review will include only published articles from different databases, and no human participants will be involved.

## Results

As of September 2025, the database search has been completed, and the organization of extracted data into summary tables is currently in progress. A narrative synthesis of the findings will follow, along with drafting the systematic review manuscript. Data extraction and synthesis are expected to be finalized by December 2025, and we plan to submit the manuscript for peer-reviewed publication by January 2026.

## Discussion

### Principal Findings

Studies have demonstrated associations between psychological factors and pain perception across various medical procedures [43,44]. Research in the field of mammography has indicated that anxiety and catastrophizing may exacerbate the experience of pain, whereas adaptive coping strategies, such as ignoring pain sensations, can mitigate pain intensity [37,40]. However, to date, no systematic review has synthesized the available evidence to provide an in-depth understanding of how psychological factors specifically influence women’s pain experience during mammography.

Therefore, it is hypothesized that the results of the selected studies will confirm the significant impact of psychological factors on pain experience. Women with higher levels of anxiety or catastrophizing are expected to report greater pain intensity compared to those with lower levels of anxiety or more effective coping strategies. Additionally, the review will address a secondary research question: the extent to which women report the intensity of pain experienced during mammography, as measured by validated questionnaires. Although supplementary, this question is expected to provide a more objective assessment of the pain experience.

### Strengths and Limitations

One of the strengths of this review is its rigorous methodology, adherence to the PRISMA-P guidelines, and the use of JBI tools to assess risk of bias, which improve the validity of the findings. Additionally, the review will offer an updated, specialized, and detailed overview of how psychological factors directly influence the pain experienced by women undergoing mammography, highlighting the importance of including psychological interventions in clinical practice and helping policymakers develop patient-centered strategies. However, some limitations should also be recognized, such as the heterogeneity of studies and measurement tools for psychological parameters, which may introduce bias, along with the restriction to English-language studies. Moreover, because of this heterogeneity, conducting a meta-analysis will not be feasible. Finally, the exclusion of gray literature, although intended to ensure methodological rigor and the inclusion of peer-reviewed studies, may have introduced publication bias and limited the comprehensiveness of the evidence base.

### Future Directions

For future research, it would be useful to explore the effectiveness of psychological interventions that could be integrated into mammography clinical practice, aiming to reduce pain perception and enhance women’s overall experience. Additionally, the present review will identify existing knowledge gaps, encouraging further studies to address them and thereby deepen the overall understanding of the topic, ultimately leading to improvements in clinical practice.

### Dissemination Plan

The findings of this review will be shared through publication in a peer-reviewed journal and presentations at international radiology and public health conferences. Additionally, patient-focused educational materials may be created to enhance awareness and participation in screening programs. Dissemination will also include professional societies, clinical guidelines, and training programs for radiologic technologists, ensuring that the results influence both practice and policy.

### Conclusions

To our knowledge, this will be the first systematic review to thoroughly examine the impact of psychological factors, such as anxiety, depression, fear, coping strategies, and others, on how women perceive and experience pain during mammography. The expected findings aim to provide evidence-based insights that inform clinical practice, support the development of targeted interventions, and reduce psychological barriers to screening participation. Ultimately, this knowledge could promote patient-centered care, increase adherence to breast cancer screening, and contribute to earlier detection and better health outcomes.

## Supplementary material

10.2196/77118 Checklist 1 PRISMA-P checklist.

## Abbreviations

JBI: Joanna Briggs Institute
PRISMA: Preferred Reporting Items for Systematic Reviews and Meta-Analyses
PROSPERO: Prospective Register of Systematic Reviews
